# Can Radiomics Predict Pathologic Complete Response After Neoadjuvant Chemoradiotherapy for Rectal Cancer? A Systematic Review and Meta-Analysis of Diagnostic-Accuracy Studies

**DOI:** 10.3390/jpm15060244

**Published:** 2025-06-10

**Authors:** Fotios Seretis, Antonia Panagaki, Stavroula Tzamouri, Tania Triantafyllou, Charikleia Triantopoulou, Dimitrios Theodorou

**Affiliations:** 1 1st Department of Propaedeutic Surgery, Hippokrateion General Hospital of Athens, 11527 Athens, Greece; stamatinat@med.uoa.gr; 2 Department of Gastroenterology, “Konstantopouleio” General Hospital of Nea Ionia, 14233 Athens, Greece; panagaki.antonia@gmail.com; 3 Department of Radiology, Evangelismos General Hospital of Athens, 10676 Athens, Greece; stavroula.tzamouri@gmail.com (S.T.); dtheodorou@med.uoa.gr (D.T.); 4 Department of Radiology, “Konstantopouleio” General Hospital of Nea Ionia, 14233 Athens, Greece; ctriantopoulou@gmail.com

**Keywords:** radiomics, MRI, rectal cancer, response, neoadjuvant, pathologic complete

## Abstract

**Background:** The rectal cancer treatment paradigm is rapidly changing with the advent of total neoadjuvant therapy and non-operative management approaches in responders. A good clinical response to neoadjuvant treatment documented by magnetic resonance imaging, endoscopy and clinical examination corresponds, to a large extent, to a pathologic complete response, as assessed in surgical specimens. **Methods:** We undertook a systematic review and meta-analysis on the MRI-based omics approach to predicting pathologic complete responses. **Results:** A total of 29 studies with relevant data available reporting on a total of 4486 patients were eligible for meta-analysis. The calculated values for the area under the curve in receiver operator curves of diagnostic accuracy for radiomics-only and radiomics-combined-with-clinical-data models were 0.80 and 0.88, respectively, for studies incorporating baseline imaging data only. The value for studies using delta radiomic data was 0.86, and those for studies using data from the post-neoadjuvant setting were 0.75 and 0.83, respectively, for the radiomics-only and radiomics-combined-with-clinical-data models. **Conclusions:** Radiomics-based prediction models for pathologic complete response assessment might further enable individualized treatment decisions to be made in patients with rectal cancer.

## 1. Introduction

Rectal cancer is a clinical entity representing unique challenges in its management. Data from randomized controlled trials have shifted treatment from the traditional scheme of neoadjuvant radiotherapy with sensitizing chemotherapy, followed by curative intent surgery with the addition of postoperative chemotherapy, toward a total neoadjuvant treatment regimen. This relatively new concept entails the administration of induction chemotherapy followed by chemoradiation (INCT-CRT) or chemoradiation followed by consolidation chemotherapy (CRT-CNCT) and the performance of radical surgery afterwards. Patients who have achieved a complete or near-complete response after completing neoadjuvant treatment may be put on a watch-and-wait management protocol with surveillance, thus potentially avoiding surgery and its associated morbidity and mortality burden. Curative intent surgery, namely total mesorectal excision (TME), is recommended for those who have achieved an incomplete response, sometimes referred to as “salvage surgery”. The recently published 5-year follow-up results of the OPRA Trial [1] support this new paradigm of treatment, demonstrating long-term organ preservation rates and no significant difference in disease-free survival after salvage surgery compared to upfront surgery. A recent consensus tried to [2] establish the definition of a near-complete clinical response after neoadjuvant (chemo)radiotherapy for rectal cancer, underlining the important role of magnetic resonance imaging (MRI) in the restaging of tumors, especially T2-weighted images (T2W) and diffusion-weighted imaging (DWI). More importantly, this consensus has proposed a certain time frame in which the tumoral response should be assessed, after which it is assigned either a complete or an incomplete response status. MRI has traditionally been used to accurately stage rectal cancer tumors, with significant implications for treatment planning as well as estimation of the risk of local as well as systemic spread of the disease. The whole landscape of rectal cancer evaluation at baseline is further complicated by an increasing body of literature suggesting that certain features of the tumor and its associated mesorectum evident on MRI, such as extramural vascular invasion, non-nodal tumor deposits, and involvement of the mesorectal fascia (MRF), may be even more important than tumor invasion depth (T stage) and nodal stage (N stage) according to the current TNM classification [3,4]. Trying to identify features on imaging linked to an increased risk of local or systemic treatment failure is a key concept, inherently linked to tumor biology. From a cancer biology perspective, it is important to clarify whether a cancerous tumor in the rectum has grown to a significant size and whether it has acquired the necessary properties to be able to involve surrounding structures (i.e., MRF), leading to local failure and local recurrence, or be able to directly invade vessels or fundamentally “survive” unchecked by the immune system in the form of tumor deposits outside of lymph nodes, thus leading to an increased risk of systemic spread [5]. MRI is an established modality that dramatically shapes decisions regarding management. According to current practice, these imaging results carry significant predictive power, as they represent a core parameter in treatment planning and individualized care for patients with rectal cancer [6].

After neoadjuvant treatment, pathologic complete response of the tumor appears to have better long-term survival outcomes than residual carcinoma in situ [7]. This process seems to be captured by MRI as well; thus, the MRI tumor regression score appears to potentially predict the pathologic complete response (PCR) after neoadjuvant treatment [8]. However, restaging tumors with MRI is also hampered by limitations, especially in mucinous tumors [9,10].

The advent of artificial intelligence and radiomics of rectal cancer in MRI creates potentially unique opportunities for enhancing diagnostic accuracy while taking into consideration the already established parameters being assessed in clinical practice daily [11]. The purpose of this systematic review is, therefore, to try to classify the current literature regarding the application of MRI radiomics according to the prognostic factors already established, and to classify imaging studies according to the MRI interrogation of tumors per se, the tumor microenvironment or tumor-related imaging findings, such as node status, etc. Special emphasis is placed on identifying whether MRI radiomics can assist in predicting responses to neoadjuvant (chemo)radiotherapy, especially when correlated to PCR [12]. The ability to predict PCR is also clinically relevant in the era of total neoadjuvant treatment (TNT) and watch-and-wait (WW) protocols [12,13]. We also conduct a meta-analysis of diagnostic accuracy, as expressed by receiver operator curves of radiomics-based and combined clinical–radiomics models for predicting the pathologic complete responses of rectal cancers after neoadjuvant treatments.

## 2. Methods

A systematic review of the literature was undertaken, including studies published until 31st of December 2024 in the PubMed and Cochrane databases, according to the Preferred Reporting Items for Systematic reviews and Meta-Analyses (PRISMA) guidelines [14]. Two independent researchers (AP) and (FS) reviewed all of the abstracts identified. Only articles in English were considered for analysis. The search terms that were used were “radiomics AND rectal cancer”, “artificial intelligence AND MRI AND rectal cancer” and “radiomics AND magnetic resonance AND rectal cancer”. The references of the articles were also manually reviewed. Only articles written in the English language were retrieved for analysis. All conflicts regarding study inclusion were resolved by a senior author (CT). The inclusion criteria were studies on the use of MRI-based radiomics for patients with rectal cancer undergoing neoadjuvant treatment that report on the ability of radiomics to predict the pathologic complete response as documented subsequently in surgical specimens. Only articles including neoadjuvant treatment and pathologic complete responses in surgical specimens were documented. Articles correlating MRI radiomics with complete pathologic responses were included in the final analysis. Details regarding the type of neoadjuvant treatment given and the time that elapsed between neoadjuvant treatment and surgery were also documented. The types of MRI sequences performed were also documented. MRI sequences performed either pre or post neoadjuvant treatment were used for radiomic feature extraction. When available, the software used for radiomic feature extraction was also documented. We purposefully did not include details on specific protocols/algorithms used for radiomics model construction from the various studies. The exclusion criteria were studies not written in English, studies with no pathologic data available, and studies in which omics diagnostic models were based on other imaging modalities such as computed tomography or ultrasound.

A flowchart of the literature search, and the identification and selection of studies for final analysis, was constructed according to the PRISMA guidelines [15].

We went on to subsequently perform a meta-analysis of the AUC values for studies fulfilling our inclusion criteria. A random effects model was used. The I^2^ statistic was used to assess the heterogeneity of the studies. For conducting the meta-analysis, including the creation of funnel plots and forest plot charts, we used the freely available software JAPS (Jeffrey’s Amazing Statistics Program) from the University of Amsterdam (Version 0.19.3).

Regarding the assessment of the methodological quality of the included studies, the QUADAS-2 tool [16] for studies reporting on the diagnostic accuracy of a diagnostic modality was used. The results were tabulated in a traffic light chart.

## 3. Results

The initial search yielded 773 articles. After the exclusion of duplicates and after the screening of abstracts for relevance, 102 articles were identified for review. After full-text review, 29 studies were selected for final analysis and are graphically depicted in Table 1 and Table 2. A PRISMA flowchart was also constructed (available in Supplementary Material S1).

Thirty-one studies were identified that reported on MRI-based radiomics models for predicting pathologic complete responses in surgical specimens of patients with rectal cancer. The majority of studies reported on patients with locally advanced rectal cancers who underwent neoadjuvant chemoradiotherapy. Table 1 includes information from the studies, including the patient demographics; number of patients included; study period; point of time in relation to neoadjuvant treatment at which MRI was performed, designated as “baseline” or “post”; time until surgery; and pathologic complete response rate on final pathology. In the vast majority of studies, an MRI scan was performed at baseline assessment and then at 6–8 weeks after the completion of neoadjuvant treatment, at which time patients were offered surgery. Regarding pathologic complete response rates, in most studies, the rate of pCR is less than 20%, in accordance with the existing literature on outcomes after “traditional neoadjuvant chemoradiotherapy”, before the advent of total neoadjuvant therapy approaches.

With regard to the MRI-based radiomics models used in the various studies, we have summarized results in Table 2. We have included the sequence of MRI used, the radiomic software used, whether the results were subject to external validation, the time frame in relation to neoadjuvant treatment for radiomic feature extraction, whether a pure radiomics model or a combined radiomics–clinical model was constructed and, finally, the test diagnostic accuracy of the model, expressed as the area under the curve (AUC) on relevant receiver operator curves.

All studies were retrospective in nature and included patients with locally advanced rectal cancer (LARC). All patients received neoadjuvant treatment, namely chemotherapy with radiotherapy. Chemotherapy regimens included FOLFOX (fluorouracil, leucovorin, oxaliplatin) or modified FOLFOX (mFOLFOX); mFOLFOX6; XELOX (oxaliplatin, capecitabine); capecitabine; and radiotherapy (RT).

Most models constructed were based on baseline T2-weighted images (T2W) with or without diffusion-weighted imaging (DWI). Sequences either pre or post neoadjuvant treatment were used. In a few studies, the delta radiomics were calculated, defined as pre–post/pre features. Most studies used PyRadiomics software for radiomics model construction. The PCR rates were expressed as percentages ranging from 15 to 81% and were sometimes expressed as the tumor regression score (TRG), according to American Joint Commission on Cancer (AJCC), with TRG0 defining the absence of a tumor and TRG1 a single cell or small groups of cells. We attempted to extract which radiomic features correlated with pCR in the included studies. However, as the radiomic parameters used in the various studies were not the same and, in many instances, not even disclosed in the reviewed manuscripts, we elected to focus on the diagnostic accuracy of the created models to predict pathologic complete responses.

The diagnostic accuracy of most studies achieved an AUC value above 0.8, which is regarded in the literature as the threshold for clinically meaningful diagnostic accuracy. Most studies achieved better AUC values when using a model combining clinical and radiomic features. Studies based on baseline-only data, post neoadjuvant-only data and delta (defined as the ratio of pre–post/pre radiomic data) were subjected separately to meta-analysis to avoid combining the diagnostic accuracies of models using data from different points in time. Combining baseline data with delta radiomics or data obtained post neoadjuvant treatment might produce misleading results.

### 3.1. Meta-Analysis of Radiomics-Only and Radiomics-Combined-with-Clinical-Data Models for pCR Prediction

We conducted a meta-analysis of the diagnostic accuracy of radiomics-only and radiomics-combined-with-clinical-data models for pCR prediction. A total of 31 studies with relevant data available reporting on a total of 4486 patients were eligible for meta-analysis.

**Figure 1:**
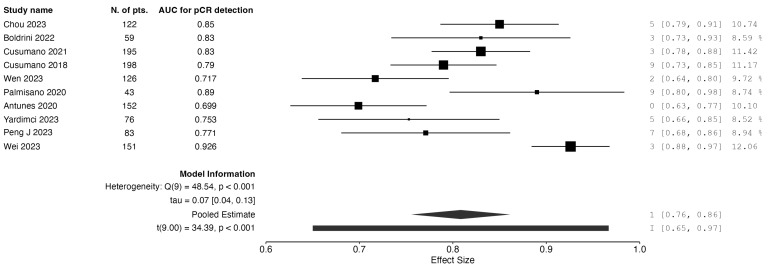
Forest plot of AUC for pCR detection: radiomics-only models from baseline data.

**Figure 2:**
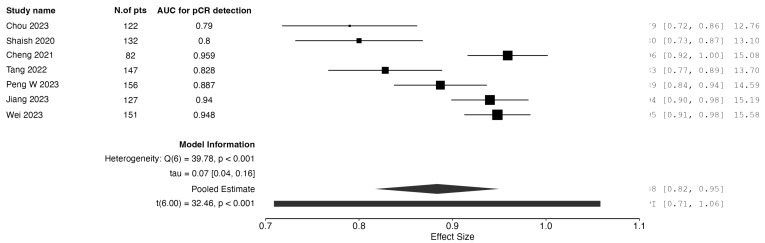
Forest plot of AUC for pCR detection: radiomics + clinical models from baseline data.

Nineteen studies reported on models based on baseline imaging data. Ten studies used radiomics-only models for pCR prediction. The calculated AUC value for radiomics-only models was 0.808. Seven studies reported on models based on radiomic data combined with clinical data. After the meta-analysis, the combined AUC value for these studies was 0.88. The results are graphically depicted as forest plot charts in Figure 1 and Figure 2, respectively.

**Figure 3:**
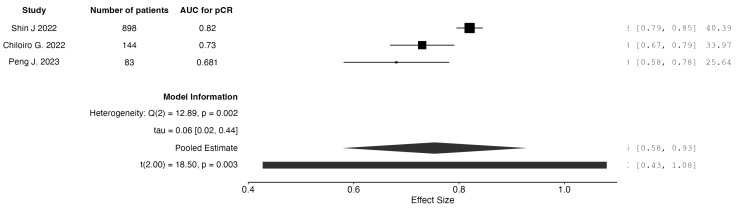
Forest plot of AUC for pCR detection: radiomics-only models from post-treatment data.

**Figure 4:**
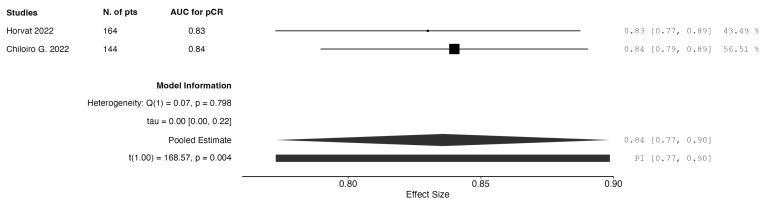
Forest plot of AUC for pCR detection: radiomics + clinical models from post-treatment data.

When analyzing studies incorporating only post-neoadjuvant treatment data, three studies reported on radiomics-only models. The pooled AUC for pCR prediction was calculated to be 0.75. Three studies reported on models combining clinical and radiomic data from the post-neoadjuvant therapy time frame. The pooled AUC was calculated to be 0.83. The results are graphically depicted as forest plot charts in Figure 3 and Figure 4, respectively.

**Figure 5:**
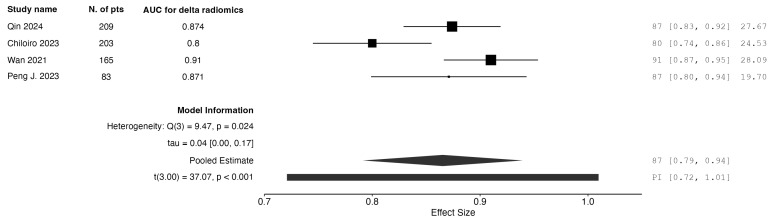
Forest plot of AUC for pCR detection: radiomics-only models using delta radiomics.

Finally, studies that reported on delta-radiomics models were also analyzed separately. Four studies reported on radiomics-only models. The pooled AUC was calculated to be 0.865. The results are graphically depicted as forest plot charts in Figure 5.

### 3.2. Risk-of-Bias Assessment—Quality of Studies

A significant degree of heterogeneity was identified for both analyses, with relevant I^2^ values of 78% for radiomics-only models and 87% for radiomics + clinical data models, of studies extracting data from baseline only. The corresponding I^2^ for studies from the post-neoadjuvant treatment setting was 83% for radiomics-only models. Regarding studies on delta radiomics, I^2^ was calculated to be 68%. This finding can be explained by the very definition of delta radiomics, which encompasses information both from the baseline and the post-neoadjuvant therapy setting and, therefore, yields increased diagnostic accuracy. All data on the risk-of-bias assessment are available in the Supplementary Materials (Supplementary Material S2–S6).

Using the QUADAS 2 criteria, we constructed a traffic light chart to visualize the quality of the studies (Table 3). The quality of the included studies was moderate at best.

## 4. Discussion

The accurate assessment of responses to the neoadjuvant treatment of rectal cancer represents a goal for all disciplines involved in rectal cancer care. MRI has been established as key in staging and prognosticating rectal cancer. MRI-based scoring systems for staging and patient stratification for local recurrence have been described [48], indicating persistent MRF invasion and extramural vascular invasion (EMVI) after neoadjuvant treatment as risk factors for local recurrence. Emerging data on intra-tumoral heterogeneity, as evaluated by diffusion-weighted imaging, appear to correlate with tumor stromal pathological heterogeneity, potentially predicting deeper invasion, pathological lymph node status and the therapeutic effects of neoadjuvant chemoradiotherapy [49].

Building upon these foundations, the omics approach to MRI has been utilized to accurately estimate tumor T stage [50], lymph node metastasis [51], EMVI [52], lateral lymph node status [53] and perineural invasion [54], features which have been consistently validated in the literature to be of prognostic value [55]. The MRI tumor regression score has been already described to predict tumor response [56]. The evolving treatment paradigm shift with the advent of total neoadjuvant treatment calls for the accurate assessment of neoadjuvant therapy response [57]. A recent international expert-based consensus attempted to standardize definitions for complete, near-complete and incomplete responses of rectal cancer to neoadjuvant treatment, including findings from digital rectal examination, MRI findings and endoscopic assessment [2].

The application of radiomics in analyzing MRI at baseline and after neoadjuvant treatment presents a new path to overcoming the limitations of classical MRI staging and restaging [58]. Radiomics-based models appear to perform moderately well in terms of their ability to predict pathologic complete responses after neoadjuvant treatment. When combined with clinical models, they appear to have even greater diagnostic accuracy, as indicated by the AUC in the ROC curves. We have demonstrated pooled diagnostic accuracy, expressed as the area under the curve, with a value of 0.833 for radiomics-only models and 0.858 for radiomics-combined-with-clinical-data models. MRI radiomics could therefore be used to select candidates thought to be complete clinical responders to successfully follow a watch-and-wait policy with rectal preservation [59].

Extracting MRI features for radiomic analysis from pre-treatment and post-treatment scans might create some concerns that pre-treatment imaging data are combined “inappropriately” with post-treatment imaging features, because the latter, by definition, indicate how the tumor responds to treatment, while the former characteristics assess the biologic behavior a priori. One might, therefore, argue that MRI-based radiomics should be separately assessed for the pre-treatment and post-treatment stages, in an attempt to use the baseline MRI scan as a predictor of the subsequent pathologic complete response. In our opinion, a primary focus of MRI-omics research in rectal cancer should be on its application in selecting candidates for non-operative approaches after neoadjuvant therapy. In other words, using MRI-based omics for predicting pathologic complete responses might correlate an excellent response on imaging to an excellent pathologic complete response. It is exactly this association of imaging with pathologic data that might be enabled by the use of the omics approach in MRI for rectal cancer. This is the reason that, although we elected to analyze studies separately using only pre-treatment data, we feel that this might not be clinically applicable, considering the fact that currently, pre-treatment data are not used to inform decisions regarding potential watch-and-wait strategies. Models using data from the baseline only yielded a pooled AUC of only 0.8, thus not being robust enough for clinical decisions. When clinical data were incorporated, the AUC rose to 0.88, thus suggesting that the mere assessment of baseline imaging data, at the present time, should not be solely used to direct patients toward potentially organ-preserving strategies. From the same point of view, when examining studies including data from the post-neoadjuvant treatment time frame, the pooled AUC was again only moderate, at 0.75, for radiomics-only models and 0.83 when radiomic data were combined with clinical data, suggesting that using diagnostic information without taking into account preoperative data is also not robust enough. On the other hand, delta radiomics appeared to have the highest pooled AUC in our study, suggesting that it is the change in certain imaging features that carries significant information of prognostic and potentially predictive value.

In addition to being a sophisticated tool for accurate staging and prognostication, the application of the omics approach in magnetic resonance-based imaging for rectal cancer brings forth the rather new concept of the “agnostic” interpretation of data from images, rather than trying to extrapolate certain characteristics from images, which has historically been found to be of prognostic value. This concept suggests that we should combine the complex biology of cancer with the way it appears on imaging, surpassing what the human brain can conceive and algorithmically process. Essentially, information contained in images is linked to cancer biology. A recently published paper from the RECIST (response evaluation in solid tumors) Working Group proposed radiomics as a precision medicine biomarker in oncology drug development and clinical care [60]. The personalization of immunotherapy by radiomics is a new concept, in line with the afore-mentioned studies [61]. This is the reason why we are currently experiencing a surge in radiomics use in cancer management, with an ever-increasing number of fields of application and modalities [62].

All studies identified in our study were retrospective in nature, thus potentially suffering from selection bias. We have demonstrated a significant degree of heterogeneity among the study results based on I^2^ statistic values above 0.83 in terms of the reproducibility of the results. Although most studies included the algorithms and analyses used for the construction of their models, their complexity limits their widespread application. Moreover, due to the relatively small number of included studies, we refrained from segregating studies according to pre/post-treatment-only status with or without the addition of clinical data, or according to delta radiomics-only data with or without clinical data. This might constitute another limitation in our study. Finally, another limitation is that in only 6 out of 31 studies was external validation of the prediction model performed.

## 5. Conclusions

Radiomics appears to be a sophisticated tool that can improve upon our current ability to predict the pathologic complete response of a tumor after neoadjuvant treatment. If the pathologic complete response can be predicted accurately by radiomics, then such imaging results, either at baseline and/or upon restaging of the tumor after neoadjuvant treatment, can guide decision-making with regard to the watch-and-wait and organ preservation approaches. However, further refinement and standardization of techniques as well as the external large-scale validation of results should be entertained before such tools can be implemented into clinical practice.

## Figures and Tables

**Table 1:** List of studies, number of patients, neoadjuvant treatment and pCR rate.

Study First Author	Year	N. pts/Stage	Neoadjuvant Regimen	Time of MRI (Baseline/Post nCRT)	Time Until Surgery	pCR Rate
Wei Q. [17]	2017–2020	151 LARC cT3-4N0Mo or cTanyN + Mo	mFOLFOX6 OR 6 XELOX OR 3-6 capecitabine OR 5FU-leucovorin + IMRT (intensity-modulated radiotherapy)	Baseline Post nCRT (5–7 weeks)	6–8 weeks	24% in first cohort, 17.6% in external validation cohort
Peng J. [18]	2017–2022	83 LARC cT3-4N0Mo or cTanyN + Mo	N/A	Baseline Post nCRT (4 weeks)	N/A	26%
Wu J. [19]	N/A	28 LARC cT3-4No or cT3-4N1-2	SCRT + mFOLFOX6	N/A	N/A	18.5%
Yardimci A. [20]	2017–2021	76 LARC cT2-4 and/or N+	RT + concurrent capecitabine	Baseline	6–10 weeks	N/A
Defeudis A. [21]	2010–2018	95 stage II/III	Chemoradiotherapy (capecitabine or 5FU or CapeOx or FOLFOX) or radiotherapy only	Baseline	N/A	N/A Pts classified as TRG1–2 Vs. TRG3–5
Lee Y. [22]	2010–2021	148 LARC cT2-4 and/or N+	Chemoradiotherapy not specified	Baseline Post nCRT	N/A	14.4% Pts classified as good/bad response
Antunes J. [23]	2009–2019	152 LARC cT3-4,N+ or threatening near structures	Long-course chemoradiotherapy	Baseline	Median of 26 days after end of nCRT	22%
Palmisano A. [24]	2013–2018	43 LARC cT2-4 and/or N+	Chemoradiotherapy (5FU + oxaliplatin + RT)	Baseline Mid-nCRT Post nCRT	N/A	25.6%
Chen H. [25]	2010–2018	39 LARC cT2-4 and/or N+	Chemoradiotherapy (not specified)	Baseline Post nCRT	N/A	23.1%
Wan L. [26]	2015–2018	165 LARC	Long-course CRT OR short-course CRT	Baseline Post nCRT	N/A	16.4%
Chiloiro G. [27]	2008–2016	203 LARC Stage II/III	CRT (5FU or CapOx or capecitabine + RT)	Baseline Post nCRT	8–12 weeks post nCRT	28.6%
Wen L. [28]	2014–2018	126 LARC Stage II/III	CRT (XELOX or mFOLFOX or FOLFOX6 +RT)	Baseline Post nCRT		22.2%
Jiang H. [29]	2012–2020	127 LARC	CRT Chemotherapy (capecitabine or capecitabine + oxaliplatin or capecitabine + 5FU) + RT	Baseline	6–8 weeks post nCRT	TRG1–2 good response, TRG3–5 bad response
Peng W. [30]	2015–2018	126 LARC Stage II/III	Long-course CRT or short-course CRT	Baseline Post nCRT	N/A	16.5% in testing cohort, 14% in validation cohort
Tang B. [31]	2017–2020	147 (59 + 88) LARC	Long-course CRT or short-course CRT	Baseline	6–8 weeks	16.9% original cohort, 13.6% validation cohort
Shin J. [32]	2009–2018	898 LARC	nCRT not otherwise specified	Baseline Post nCRT	N/A	21%
Cheng Y. [33]	2014–2018	193 LARC	nCRT (mFOLFOX6/CapeOX + RT)	Baseline	6–8 weeks	16%
El Homsi M. [34]	2013–2019	98 LARC	nCRT (capecitabine/FOLFOX +RT)	Baseline	80 days from nCRT to surgery	16.3%
Cusumano D. [35]	2008–2014	198 LARC	nCRT (not specified)	Baseline	6–8 weeks	27% and 28% in the two cohorts
Shaish H. [36]	2009–2019	132 LARC	nCRT (data not available on all patients)	Baseline	N/A	15%
Cui Y. [37]	2012–2016	186 LARC	nCRT (capecitabine + RT)	Baseline	9 weeks	16.7%
Petkovska I. [38]	2011–2015	102 LARC	nCRT (capecitabine + RT)	Baseline	85 days average	19%
Boldrini L. [39]	2017–2018	221 LARC	nCRT chemotherapy (capecitabine/5-FU/CapOx) + RT	Baseline	8–12 weeks	16.9%
Chiloiro G. [40]	2010–2019	144 LARC	nCRT chemotherapy (capecitabine/5-FU/CapOx) + RT	Post nCRT	8 weeks	81%
Cusumano D. [41]	2008–2014	195 LARC	nCRT chemotherapy (capecitabine/5-FU/CapOx) + RT	Baseline	6–8 weeks	22%, 25%
Filitto G. [42]	2018–2020	43 LARC	nCRT (capecitabine + RT)	Baseline	N/A	41% (TRG0, TRG1 as per AJCC)
Li Z. [43]	2016–2019	80 LARC	nCRT (capecitabine + RT)	Baseline Post nCRT	8–10 weeks	18.75%
Horvat N. [44]	2012–2016	164 LARC	nCRT		N/A	18% in first cohort, 16% in second cohort
Chou Y. [45]	2010–2019	133 LARC	nCRT (uracil-tegafur + mitomycsin + RT)	Baseline	6–8 weeks	26%
Qin S. [46]	2013–2022	209 LARC	nCRT (XELOX or capecitabine + RT)	Baseline Post nCRT	6–8 weeks	21.1%
Crimi 2024 [47]	2016–2022	102 Stage II, III	nCRT (capecitabine or 5-FU chemotherapy + RT_	Baseline	N/A	23.5%

Abbreviations: LARC—locally advanced rectal cancer; nCRT—neoadjuvant chemoradiotherapy; RT—radiotherapy; TRG—tumor regression score; LCRT—long-course radiotherapy; SCRT—short-course radiotherapy.

**Table 2:** List of studies, software used for radiomic analysis and area under the curve (AUC) for pCR prediction.

Study First Author	MRI Sequence	Radiomics Software Used	External Validation	Model Created (Radiomics OR Radiomics + Clinical)	Time of Radiomic Extraction in Relation to nCRT	Maximum AUC Achieved: Radiomics	Maximum AUC Achieved: Radiomics + Clinical
Wei Q. [17]	T2WI DWI	PyRadiomics	Yes	Radiomics Radiomics + Clinical	Pre Post	0.926 initial cohort, 0.829 in external validation	0.948 in initial cohort, 0.872 in external validation
Peng J. [18]	T2WI DWI T1WI + C	PyRadiomics	No	Radiomics	Pre Post Delta (Pre–Post/Pre)	Pre 0.771 Post 0.681 Delta 0.871 Combined	0.907
Wu J. [19]	N/A	PyRadiomics	No	Radiomics + clinical	Pre + Delta	N/A	N/A
Yardimci A. [20]	T2WI	PyRadiomics	No	Radiomics Radiomics + clinical	Pre	0.753	0.767
Defeudis A. [21]	T2WI	PyRadiomics	Yes	Radiomics	Pre	0.90 initial cohort manual-0.61 external validation	N/A
Lee Y. [22]	T2WI	PyRadiomics	No	Radiomics Radiomics + Clinical	Pre Post	0.74	0.79
Antunes J. [23]	T2WI	MATLAB R2018a	No	Radiomics	Pre	0.699	N/A
Palmisano A. [24]	T2WI DWI T1WI + C	Olea Medical Software, La Ciotat, France	No	Radiomics	Pre Post	0.89	N/A
Chen H. [25]	T2W	IBEX	No	Radiomics	Pre Post	Accuracy 92%	N/A
Wan L. [26]	T1WI T2WI DWI	Radcloud Platform	No	Radiomics	Delta	0.91	N/A
Chiloiro G. [27]	T2WI DWI	Moddicom	No	Radiomics + Clinical	Delta	0.80	0.80
Wen L. [28]	T2WI	MaZda	No	Radiomics + Clinical	Pre Post	Pre 0.717 Post 0.805 Delta 0.724	0.852
Jiang H. [29]	T2WI DWI	PyRadiomics	No	Radiomics + Clinical	Pre	N/A	DWI + clinical 0.87 T2W + clinical 0.81 Fusion + clinical 0.94
Peng W. [30]	T2WI DWI	Pyradiomics	No	Radiomics + Clinical	Pre Post	N/A	Pre-treatment_ + post-treatment combined 0.887
Tang B. [31]	T2WI	Moddicom	Yes	Radiomics	Pre	N/A	0.831 initial 0.828 validation
Shin J. [32]	T2WI ADC T2WI + DWI fused	PyRadiomics	No	Radiomics	Post	0.82	-
Cheng Y. [33]	T1W, T2W and T2FS	Pyradiomics	No	Radiomics + Clinical	Pre	N/A	0.959
El Homsi M. [34]	T2W	N/A	No	Radiomics	Pre	0.9	-
Cusumano D. [35]	T2W	Moddicom	Yes	Radiomics	Pre	0.79	-
Shaish H. [36]	T2W	Pyradiomics	No	Radiomics + Clinical	Pre	N/A	0.8
Cui Y. [37]	T2W c-T1W ADC	Analysis Kit software, GE Healthcare, Wuxi, China	No	Radiomics + Clinical	Pre	N/A	0.94 training set 0.944 validation set
Petkovska I. [38]	T2W DWI	Computational Environment for Radiological Research (CERR) software	No	Radiomics + Anatomic	Pre	N/A	0.75
Boldrini L. [39]	T2W	Moddicom platform	Yes	Radiomics	Pre	0.83	-
Chiloiro G. [40]	T2W DWI	N/A	No	Radiomics + Clinical	Post	0.73	0.84
Cusumano D. [41]	T2W	Moddicom	No	Radiomics	Pre	0.83	-
Filitto G. [42]	T2W	Pyradiomics	No	Radiomics	Pre	Precision 0.7	-
Li Z. [43]	T2W	Pyradiomics	No	Radiomics	Pre Post	0.94	-
Horvat N. [44]	T2W	N/A	Yes	Radiomics + Clinical	Post	N/A	0.83
Chou Y. [45]	T2W	Pyradiomics	No	Radiomics, Radiomics + Clinical	Pre	0.85	0.79
Qin S. [46]	T2W DWI	uAI Research Portal Software Version:1.1	No	Radiomics	Delta	0.874	N/A
Crimi 2024 [47]	T2W	Trace4Research Platform	Yes	Radiomics	Pre	0.73	N/A

Abbreviations: T2W—T2-weighted MRI; DWI—diffusion-weighted MRI.

**Table 3:** QUADAS 2 traffic light chart.

Study	Risk of Bias				Applicability Concerns		
	Patient Selection	Index Test	Reference Standard	Flow and Timing	Patient Selection	Index Test	Reference Standard
Wei [17]	☺	☺	☺	☺	☺	☺	☺
Peng J. [18]	☺	☺	☺	☺	☺	☺	☺
Yardimci [20]	☹	☹	☺	☺	☹	☺	☹
Defeudis [21]	☹	☹	☺	☺	☹	☺	☺
Lee Y. [22]	☹	?	☺	☹	☹	☺	☺
Antunes [23]	☹	?	☹	☺	☹	?	☹
Palmisano [24]	☺	☺	☺	☺	☺	☺	☺
Wan [26]	☹	?	☺	☺	☹	?	☺
Chiloiro [27]	☺	?	☺	☺	☺	☺	☺
Wen [28]	☹	☺	☺	☹	☹	☺	☺
Jiang [29]	☺	☹	☺	☺	☺	☺	☺
Peng W. [30]	☺	☹	☺	?	☺	☹	
Tang [31]	☺	?	☺	?	☺	☹	
Shin [32]	?	☹	☹	?	?	☺	
Cheng [33]	☺	?	☹	?	☺	☹	☹
El Holmsi [34]	☺	☹	☺	☹	☺	☹	
Cusumano [35]	☹	☹	☹	?	☹	☺	☺
Cusumano 2021 [41]	☺	?	☹	?	☺	☹	☺
Shaish [36]	☺	☹	?	☺	☺	☹	☹
Cui [37]	☺	?	?	☺	☺	☹	
Petkovska [38]	?	?	?	☺	?		☺
Boldrini [39]	☺	☹	?	☺	☺	?	☹
Chiloiro [40]	☺	?	?	☹	☺	?	☹
Filitto [42]	☺	☹	?	☹	☺	?	☺
Li [43]	?	?	☺	☺	?	?	☺
Horvat [44]	☺	?	☺	☹	☺	☹	☺
Chou [45]	☺	☹	?	☹	☹	☹	☺
Qin [46]	☺	?	?	☺	☺	?	☹
Crimi [47]	☺	?	☹	?	☺	?	☹

## Supplementary Materials

The following supporting information can be downloaded at https://www.mdpi.com/article/10.3390/jpm15060244/s1, Supplementary Material S1: PRISMA flowchart, Supplementary Material S2: Table of statistical analyses for radiomic-only based models, Supplementary Material S3: Table of statistical analyses for radiomic+ clinical based models for baseline data, Supplementary Material S3: Table of statistical analyses for radiomic+ clinical based models for baseline data, Supplementary Material S4: Table of statistical analyses for radiomic-only models from post-neoadjuvant treatment only data, Supplementary Material S5: Table of statistical analyses for radiomic+ clinical data-based from post-neoadjuvant treatment only data, Supplementary Material S6: Table of statistical analyses for Radiomic-only based models from delta radiomic data.
