# Supplementary material for: Can Radiomics Predict Pathologic Complete Response After Neoadjuvant Chemoradiotherapy for Rectal Cancer? A Systematic Review and Meta-Analysis of Diagnostic-Accuracy Studies
Source: J Pers Med. 2025 Jun 10;15(6):244. doi: 10.3390/jpm15060244 (PMC12193717; doi:10.3390/jpm15060244)
Supplement: Supplementary file 1 [file jpm-15-00244-s001.zip › jpm-3555123-supplementary.pdf]

Supplementary material

1. Supplementary material S1
   PRISMA flowchart

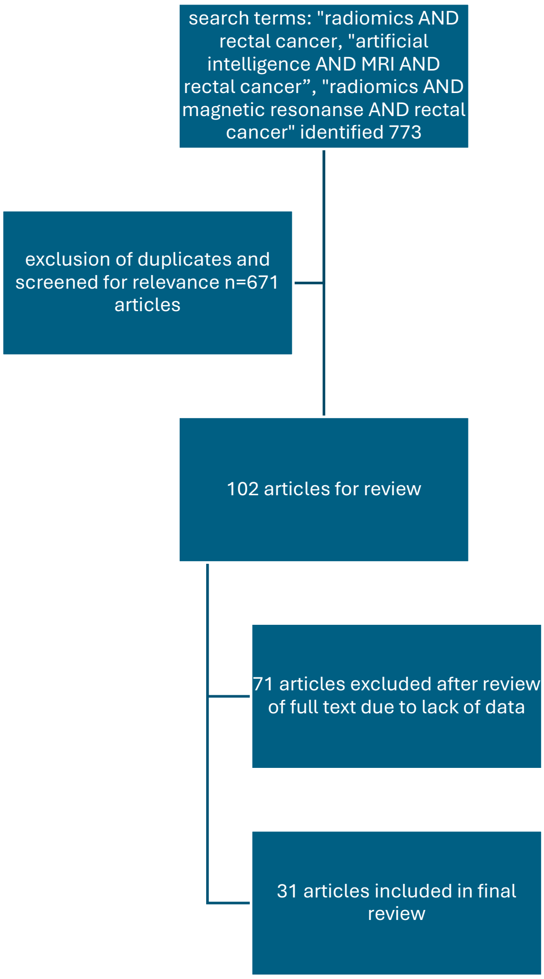

2. Supplementary material S2

Radiomic-only based models for baseline data

Pooled Effect Size Test

Estimate	Standard Error	t	df	p
0.808	0.024	34.388	9.000	< .001

Meta-Analytic Estimates

		95% CI		95% PI	
	Estimate	Lower	Upper	Lower	Upper
Effect Size	0.808	0.755	0.862	0.650	0.967

Pooled Effect Size Test

Estimate	Standard Error	t	df	p
I^2	78.579	53.471	93.319	

Note. 9 observations were ommited due to missing values.

3. Supplementary material S3

Radiomic+clinical based models for baseline data

Pooled Effect Size Test

Estimate	Standard Error	t	df	p
0.884	0.027	32.461	6.000	< .001

Meta-Analytic Estimates

		95% CI		95% PI	
	Estimate	Lower	Upper	Lower	Upper
Effect Size	0.884	0.817	0.950	0.709	1.058
I^2	87.345	68.238	97.516		

Note. 12 observations were ommited due to missing values.

4. Supplementary material S4

Radiomic-only models from post-neoadjuvant treatment only data

Pooled Effect Size Test

Estimate	Standard Error	t	df	p
0.754	0.041	18.497	2.000	0.003

Meta-Analytic Estimates

		95% CI		95% PI	
	Estimate	Lower	Upper	Lower	Upper
Effect Size	0.754	0.578	0.929	0.427	1.080
I^2	83.083	37.514	99.574		

Note. 2 observations were ommited due to missing values.

5. Supplementary material S5

Radiomic+clinical data-based models from post-neoadjuvant treatment only data

Pooled Effect Size Test

Estimate	Standard Error	t	df	p
0.836	0.005	168.566	1.000	0.004

Meta-Analytic Estimates

		95% CI		95% PI	
	Estimate	Lower	Upper	Lower	Upper
Effect Size	0.836	0.773	0.899	0.773	0.899
I^2	0.000	0.000	98.505		

Note. 3 observations were ommited due to missing values.

6. Supplementary material S6

Radiomic-only based models from delta radiomic data

Pooled Effect Size Test

Estimate	Standard Error	t	df	p
0.865	0.023	37.070	3.000	< .001

Meta-Analytic Estimates

		95% CI		95% PI	
	Estimate	Lower	Upper	Lower	Upper
Effect Size	0.865	0.791	0.940	0.721	1.010
I^2	68.285	0.000	97.612		
